# Impacts of census differential privacy for small-area disease mapping to monitor health inequities

**DOI:** 10.1126/sciadv.ade8888

**Published:** 2023-08-18

**Authors:** Yanran Li, Brent A. Coull, Nancy Krieger, Emily Peterson, Lance A. Waller, Jarvis T. Chen, Rachel C. Nethery

**Affiliations:** ^1^ Department of Biostatistics, Harvard T.H. Chan School of Public Health, Boston, MA, USA.; ^2^ Department of Social and Behavioral Sciences, Harvard T.H. Chan School of Public Health, Boston, MA, USA.; ^3^ Department of Biostatistics and Bioinformatics, Emory Rollins School of Public Health, Atlanta, GA, USA.

## Abstract

The U.S. Census Bureau will implement a modernized privacy-preserving disclosure avoidance system (DAS), which includes application of differential privacy, on publicly released 2020 census data. There are concerns that the DAS may bias small-area and demographically stratified population counts, which play a critical role in public health research, serving as denominators in estimation of disease/mortality rates. Using three DAS demonstration products, we quantify errors attributable to reliance on DAS-protected denominators in standard small-area disease mapping models for characterizing health inequities. We conduct simulation studies and real data analyses of inequities in premature mortality at the census tract level in Massachusetts and Georgia. Results show that overall patterns of inequity by racialized group and economic deprivation level are not compromised by the DAS. While early versions of DAS induce errors in mortality rate estimation that are larger for Black than non-Hispanic white populations in Massachusetts, this issue is ameliorated in newer DAS versions.

## Teaser

2020 census privacy procedures yield population data suitable for use in small-area studies of disease and health inequities.

## INTRODUCTION

To release statistics about populations without violating privacy, the U.S. Census Bureau announced in 2018 that it would implement differential privacy on publicly released data products derived from 2020 decennial census (DC) data (*1*). Reporting of statistics at population scale is insufficient to ensure the privacy of individuals (*2*). Differential privacy is a mathematical framework for providing a provable and quantifiable amount of privacy protection (*3*). A mechanism/method/algorithm that satisfies differential privacy as a privacy definition often introduces noise to a target statistic. While different definitions of differential privacy exist, it generally ensures that statistics reported at specified population scales do not change substantially when a single record is included/excluded. The Census Bureau will adopt a definition of differential privacy known as zero-concentrated-differential privacy (*4*, *5*) and use an algorithm satisfying this definition to infuse random noise into census tabulations at six different nested geolevels (nation, state, county, tract, block group, and block) (*6*, *7*). Because the same amount of noise is injected for tabular statistics within the geolevels, the noise injected into small counts is relatively larger than that applied to larger counts (*8*, *9*).

The Census Bureau’s use of differential privacy as a component of its 2020 Disclosure Avoidance System (DAS) was introduced in response to a report showing that its previous methods permitted larger than expected risks of person re-identification (*10*). Differential privacy represents a departure from the disclosure avoidance procedures applied to census data in the past to generate the publicly released census tabulations, which included data suppression and/or “data swapping” (*11*, *12*). Some scholars have voiced concerns about the potential impacts of the 2020 census DAS, including differential privacy-related noise injection and the necessary postprocessing steps, on public policy and social science research and on the redistricting process, which critically rely upon census data (*1*, *13*, *14*). The *Harvard Data Science Review* recently released a special issue to document, contextualize, and assess the U.S. Census Bureau’s adoption of differential privacy and presented discussions with key stakeholders about the decision (*15*). Within this issue is a guide for researchers on responsible use of 2020 public release DC products (*16*).

Monitoring of social and spatial patterns of disease, a fundamental component of public health research and practice, also requires accurate population counts to serve as the denominator for estimation of disease rates. These population denominators are most often obtained from census products. In particular, disease monitoring typically relies on demographically stratified small-area disease rates (and associated population denominators), which are analyzed to identify and intervene on populations at highest risk. Small-area measures are central to these efforts because populations within small areas tend to be more homogeneous than in larger areas (reflecting impacts of present and past residential racialized and economic segregation and housing costs) (*17*–*21*), providing a differential between the socioeconomic and environmental characteristics of areas studied that may aid in detecting relationships between these variables and health (*22*). Thus, the potential exaggerated effects of differential privacy on census tabulations for small populations are of particular concern to the public health community. However, little research to date has investigated the potential influence of the DAS on the accuracy of small-area public health studies.

Although the U.S. Census Bureau will solely release the DAS-protected 2020 census tabulations, they have published several DAS “demonstration products” to enable the research community to evaluate and comment on its potential impacts (*23*–*25*). In each of these demonstration products, a variant of the proposed DAS procedures was applied to 2010 DC data. The variants represent sequential refinements of the DAS implementation over time. Through comparisons with the original 2010 public release census data, these products enable researchers and practitioners to assess whether and how the DAS might unintentionally induce systematic discrepancies in reported census statistics or in analyses that use them.

Few studies have leveraged the demonstration products to formally assess the potential 2020 DAS impacts in public health applications. Even fewer have focused on the consequences of using differentially private population counts in modeling of small-area disease rates for identifying health trends and monitoring disparities. One recent study compared county-level estimates of 2010 racialized group mortality rates produced using the original 2010 census population counts and the first of the Census Bureau’s demonstration products (*1*). This work estimated county-level rate differences for rates constructed with the original and demonstration product denominators and summarized the rate differences across county urbanicity strata. Using a similar approach but reporting county-level absolute percent errors in mortality rate estimates (comparing demonstration product versus original denominators), another study investigated the extent to which differential privacy could distort county-level coronavirus disease 2019 (COVID-19) mortality rates by age-sex/racialized groups (*26*). They used the second of the demonstration products and concluded that DAS-induced errors in COVID-19 mortality rates were larger for non-white racialized groups (though their use of absolute errors precludes assessment of directionality of errors). Another study compared estimates of premature mortality rates aggregated by racialized group and by census tract quintile of inequality across Massachusetts using denominators from the 2010 census versus the first DAS demonstration product. Although census tract inequality measures were used to create strata (each stratum included >200 census tracts), all mortality rates were computed and compared in aggregate for the strata, i.e., the study did not evaluate DAS impacts on small-area rate estimates. They concluded that, for these heavily aggregated metrics, the 2020 DAS procedures may have little impact on estimates of health inequities (*27*). Given the limited nature of this literature, numerous gaps remain. For instance, none of these studies have (i) examined and compared different DAS versions’ impacts for estimation of disease rates for small areas (smaller than counties) commonly used for health disparities analysis in public health practice, (ii) used the most recently released demonstration product that likely better reflects the final 2020 DAS procedures, nor (iii) conducted simulation studies to more formally quantify biases introduced by the DAS.

Therefore, in this paper, we leverage three demonstration products, including the most recently released one at the time of writing, to evaluate the performance of small-area disease mapping models using DAS-affected denominators versus original 2010 DC denominators, with an emphasis on accurate characterization of health inequities. Potential biases are illustrated using a pseudo-simulation study and real data analyses of racialized disparities in premature mortality at the census tract level in Massachusetts (MA) and Georgia (GA). Our results may help public health researchers and practitioners to determine whether the 2020 DAS-affected publicly released products will yield reliable and actionable results when used for essential public health tasks.

## RESULTS

### Case study populations

**Fig. 1.**
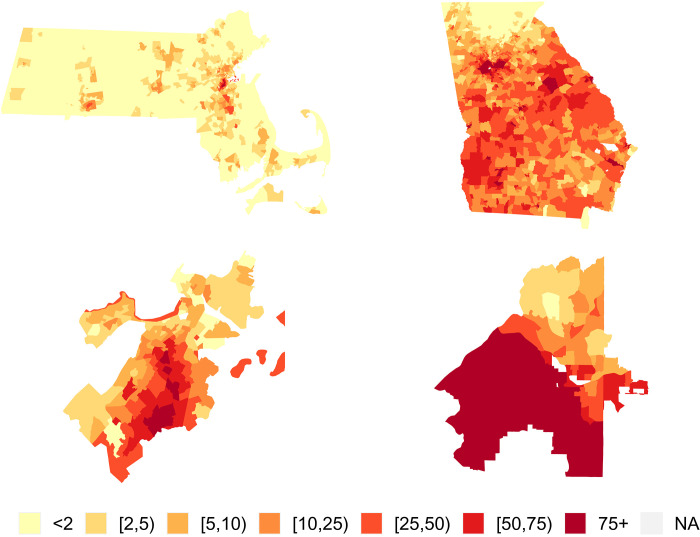
Maps of 2010 census tract–level percent Black in the study areas. Maps display the distribution across Massachusetts (**top left**), Georgia (**top right**), Boston (**bottom left**), and Atlanta (**bottom right**).

Here, we place special emphasis on evaluating the impacts of the 2020 DAS on measures of Black versus non-Hispanic white (NHW) health inequities. Given this focus, the states of MA and GA were selected as case studies because they allow us to observe the impacts of the DAS in the context of a small and highly concentrated Black population (MA) and in a relatively large and diffuse Black population (GA) (Fig. 1). Individuals identifying as Black alone made up 12.6% of the U.S. population per the 2010 DC, as compared to 30.5% of the GA population and 6.6% of the MA population. Moreover, the Black population in MA is highly concentrated in and around the Boston area, while in GA a substantial portion of the population in most of the state’s census tracts identify as Black (Fig. 1).

### Population count data

The original 2010 DC publicly released data, which are used as a “baseline” in our study, were subject to a disclosure avoidance procedure known as data swapping by the Census Bureau. In this procedure, records were swapped between geographies for households deemed to have similar characteristics before constructing publicly released tabulations (*11*, *12*). The precise implementations of data swapping, used for many decades by the Census Bureau, are not fully documented and have changed over time (*12*). Little or no research has addressed how data swapping affects accuracy of population counts or of subsequent analyses using them. Thus, the original 2010 DC public release data are themselves likely affected by data privacy–related errors; however, because they are the current gold standard, they represent an important baseline for comparison to the DAS demonstration products (described below). They have been used as such in many recent papers (*1*, *13*, *14*, *27*) and in the Census Bureau’s own assessments of the DAS (*7*, *28*).

To help data users study impacts of the proposed 2020 DAS, the Census Bureau has released a set of demonstration data products in which potential variants of the 2020 DAS (including both differential privacy algorithms and subsequent postprocessing steps) have been applied to the 2010 census data instead of the original data swapping procedure. Together, the original 2010 DC data and the demonstration products enable comparisons of the population counts themselves and the results of analyses that rely on them. Here, we use three of the demonstration products to evaluate how using DAS-protected population counts to model disease rates might bias our results if the true population counts are those from the 2010 DC (see the Discussion section for a discussion of the limitations of the use of DC population counts as a baseline and implications for our findings). In both the simulations and real data analysis, we use census tract–level population count data for each of MA and GA from the Demographic and Housing Characteristics File corresponding to each of the following four Census Bureau data products:

1) Original 2010 DC Data (DC): 2010 population counts with the standard data swapping procedures applied (*29*);

2) Demonstration product released in October 2019 (DP19): 2010 population counts with the first version of Census Bureau’s DAS procedure applied (*23*, *30*);

3) Demonstration product released in May 2020 (DP20): 2010 population counts with the second version of Census Bureau’s DAS procedure applied (*24*, *31*);

4) Demonstration product released in August 2022 (DP22): 2010 population counts with the most recent version (at the time of writing) of Census Bureau’s DAS procedure applied (*25*, *32*).

At the time of writing, the only existing demonstration product that includes a Demographic and Housing Characteristics File but is not considered in our analyses is the demonstration product released in March 2022. We excluded this demonstration product because, following its release, the Census Bureau announced that they had discovered an error in the code used to apply the DAS, resulting in unintended specifications of certain parameters in the differential privacy algorithm (*33*). These errors were corrected in the August 2022 demonstration product.

The differences between the demonstration products are described in detail in the “Differences in DAS demonstration products” section. Briefly, the DP19 and DP20 demonstration products use identical implementations of differential privacy but distinct postprocessing procedures. In response to stakeholder feedback, the differential privacy algorithm parameters were modified in the DP22 product to increase accuracy. Specifically, one of the key quantities controlling the accuracy privacy-loss trade-off in the differential privacy algorithm, known as the privacy-loss budget, was increased, yielding greater accuracy while weakening privacy guarantees.

### Inequities in premature mortality using various population data products

Data collection and processing are described only briefly here, with more detail provided in the “Data collection and processing” section. The outcome of interest of our study is premature mortality (death before 65 years old), which is an important and widely used metric in health inequities studies. We focus on inequities in risk by racialized group, comparing Black and NHW populations, and by socioeconomic status. We obtained racialized group–specific counts of premature deaths by census tract for MA (2010) and GA (2008–2012). Using each of the four population count data sources described above, we construct age-standardized premature mortality ratios (SMRs) for both the Black and NHW populations in each MA and GA census tract using the indirect standardization method (*34*). The census tract– and racialized group–specific observed premature mortality count serves as the numerator of the SMR. An expected premature mortality count for the census tract and racialized group, computed based on its population size and age distribution, serves as the denominator of the SMR. These expected counts are generated separately using each of the four population count data sources. We compare the expected counts constructed from the DC, used here as the baseline, with those obtained using DP19, DP20, and DP22, which have the DAS applied.

Figure S3 shows a scatterplot of the racialized group–stratified census tract expected premature mortality counts from the DC versus DP19, DP20, and DP22 for each state. From this figure, it is clear that in MA, Black expected counts are generally much smaller than the NHW counts, while in GA the distributions are more similar across racialized groups. In both states and for both racialized groups, the DP19 data are slightly noisier than the DP20 data (relative to DC), which are substantially noisier than the DP22 data.

**Fig. 2.**
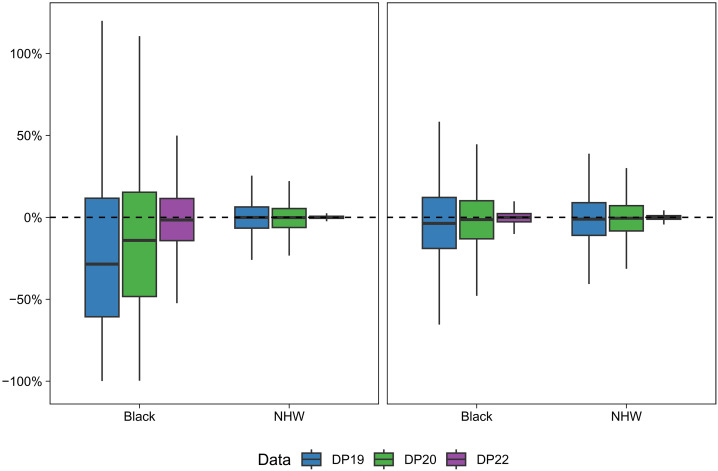
Percent difference in age-standardized population denominators from each of the DAS demonstration products and the 2010 DC. Boxplots show the percent difference in census tract expected premature mortality counts for 2010 created from the U.S. Census Bureau DAS demonstration products released in 2019 (DP19), 2020 (DP20), and 2022 (DP22), relative to original 2010 DC-based expected counts, for Black and NHW populations in Massachusetts (**left**) and Georgia (**right**).

In Fig. 2, we plot the percent difference in each of the DP19, DP20, and DP22 census tract expected premature mortality counts versus the DC analogs, stratified by racialized group (numeric summaries of these differences are provided in table S1). For the NHW population, the distributions of percent differences are narrow and centered around zero for all demonstration products and for both states. This is likely a result of the large NHW populations in most census tracts, leading to smaller relative errors injected by differential privacy. In addition, with each newly introduced demonstration product, the variability in the percent differences declines, indicating that refinements of the DAS over time are leading to near convergence of the DAS-affected and DC denominators. For the Black population, the story differs by state. In GA, which has a larger and more diffuse Black population, the magnitudes and trends in percent differences mimic those described for the NHW population. However, in MA where the Black population is relatively small and concentrated, the distribution of percent differences is much wider than for NHW. Moreover, for DP19 and DP20, the distribution of percent differences for Black populations is centered well below zero, indicating that Black expected counts tend to be underestimated in the earlier DAS variants, relative to the DC. To more thoroughly characterize this underestimation, we present in table S2 the percent of expected counts that are underestimated for each demonstration product relative to the DC, and in table S3 the percent of expected counts that are zero and that are <5 for each data source. In DP22, with the increased privacy-loss budget, the distribution of percent differences for Black populations in MA remains considerably wider than for NHW but is centered around zero.

To study the impact of the different denominators in assessing disparities in premature mortality, we fit standard Bayesian disease mapping models to the census tract–level SMRs stratified by racialized group from each of the four denominator data sources separately (see the “Real data analysis models” section). We examine their associations with racialized group and an area-level measure of economic deprivation—census tract proportion below the poverty line—extracted from the 2008–2012 5-year American Community Survey data, which is estimated for the census tract as a whole and is not race-specific. Premature mortality rate ratio estimates and 95% Bayesian credible intervals from models fit separately for MA and GA are presented in Table 1. The racialized group variable is a group-level binary indicator of Black (versus NHW) such that premature mortality rate ratios >1 for this variable indicate that, on average, census tract premature mortality rates for Black populations are higher than rates for NHW populations conditional on proportion of the census tract in poverty. The premature mortality rate ratio estimate for the racialized group variable is >1 and statistically significant for each state and for the models fit with each of the four denominator data sources. Across all denominator data sources, the estimated racialized group inequity is larger in MA than in GA, with Black premature mortality rates around 16% larger than NHW rates in MA and around 8% larger than NHW rates in GA (conditional on poverty). Proportion of census tract residents in poverty is also associated with higher premature mortality rates in the models for both states and using each denominator data source. Again, the magnitude of the inequity is larger in MA than in GA, with a standard deviation increase in proportion in poverty associated with around a 33% increase in premature mortality rate in MA as compared to a 14% increase in GA. Inferences about patterns of health disparities from the models using the four different denominator sources are identical in all cases, with only very minor differences in the point estimates (and even these may be attributable to randomness in the Bayesian posterior sampling).

**Table 1.** Estimated measures of inequities in premature mortality from real data analyses using different denominator data sources. Mortality rate ratio estimates (95% credible intervals) are shown from analyses of real premature mortality data from Massachusetts, 2010, and Georgia, 2008–2012, using each of the four denominator data sources: the 2010 DC and the U.S. Census Bureau DAS demonstration products released in 2019 (DP19), 2020 (DP20), and 2022 (DP22).

		Denominator data sources			
		DC	DP19	DP20	DP22
Massachusetts	Intercept	1.06 (1.04, 1.09)	1.06 (1.04, 1.09)	1.06 (1.04, 1.09)	1.06 (1.04, 1.09)
	Racialized group	1.16 (1.06, 1.29)	1.17 (1.06, 1.29)	1.16 (1.05, 1.28)	1.17 (1.07, 1.29)
	Poverty	1.33 (1.28, 1.38)	1.33 (1.28, 1.38)	1.32 (1.28, 1.37)	1.33 (1.29, 1.38)
Georgia	Intercept	1.82 (1.80, 1.84)	1.84 (1.82, 1.87)	1.83 (1.81, 1.85)	1.82 (1.80, 1.84)
	Racialized group	1.08 (1.06, 1.11)	1.08 (1.05, 1.10)	1.08 (1.05, 1.10)	1.08 (1.05, 1.10)
	Poverty	1.14 (1.12, 1.16)	1.14 (1.12, 1.17)	1.14 (1.12, 1.16)	1.14 (1.12, 1.16)

### Simulation study

In addition to the real data analysis, we conduct simulation studies to formally assess the magnitude of biases induced in estimates of health inequities due to using the DAS-protected denominators in standard models (relative to the 2010 DC denominators, which serve as our baseline). We design simulations so that the synthetic data resemble the real case study data for MA and GA described in the previous section, and all data generation and analyses are performed separately, but analogously, for the two states. We structure our simulated outcomes to mimic real patterns in premature mortality. Black and NHW synthetic premature mortality counts are generated for each census tract within a given state following the model form used in the real data analyses (see the “Real data analysis models” section), using the 2010 DC expected premature mortality counts as the true denominator and the real covariate data (racialized group indicator and proportion of census tract residents in poverty). See the “Data generating process” section for more details on the data generating process. We then fit models to the simulated data using the DP19, DP20, and DP22 expected counts, but otherwise correctly specified. We repeat this for 100 simulated datasets. To investigate the performance of the models fit with the different denominator data sources, we evaluate the distribution of the estimated model coefficients and the model-based SMR estimates, relative to the known true values of these quantities.

**Fig. 3.**
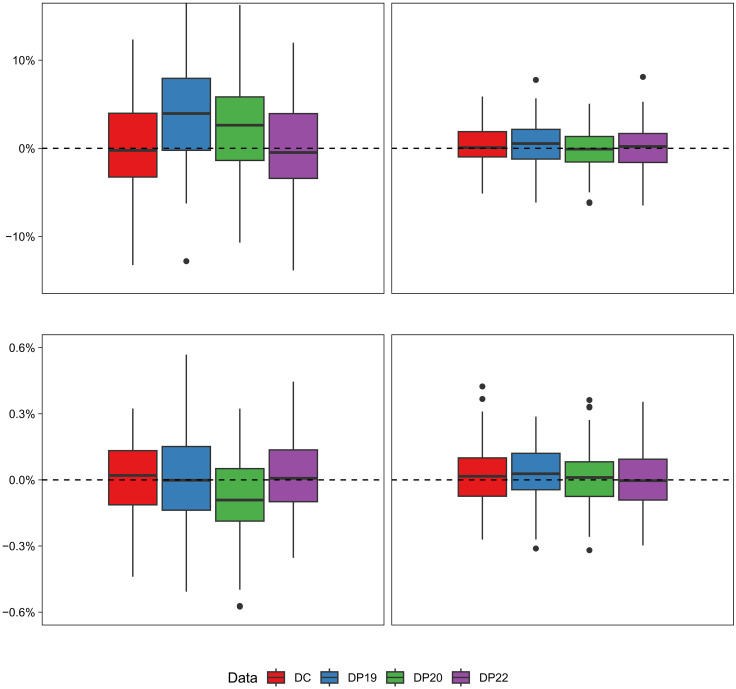
Simulation results: Bias in estimated inequity model coefficients using different denominator data sources. Boxplots represent the distribution of bias in estimates of the Black versus NHW (**first row**) and percent poverty (**second row**) coefficients over 100 simulations, with simulated data mimicking patterns of premature mortality in Massachusetts (**left column**) and Georgia (**right column**) and models fit using each of the four denominator data sources: the 2010 DC and the U.S. Census Bureau DAS demonstration products released in 2019 (DP19), 2020 (DP20), and 2022 (DP22). Data were generated using DC as the true denominator data.

We compute the simulated bias of the coefficient estimates from models fit using each of the four sets of expected counts to demonstrate how the use of DAS-protected denominators in model fitting (when DC denominators are the “true” denominators in the data generating process) affects assessment of high-level patterns and comparisons of risks across groups. The percent bias in the coefficient estimates from each simulation is summarized in boxplots in Fig. 3 (raw biases, not on the percent scale, are summarized in table S4). For each coefficient, we also report the percent of simulations in which the estimated 95% credible interval covers the true parameter value (this metric is often called the “coverage”) in table S5. Both the racialized group and poverty coefficient parameters are estimated with little to no bias, on average, when using the DC denominators (the “true” denominators used to generate the data) for model fitting. In GA, all the demonstration product denominators perform comparably to the DC denominators for estimating both the coefficient parameters. On the other hand, for MA, we can observe that using the DP19 and DP20 denominators in the model fitting leads to overestimation of the racialized inequity parameter, with average percent biases of 3.862% and 2.579%, respectively. This is likely a result of the systematic underestimation of the DAS-protected denominators for Black populations in MA (Fig. 2), leading Black premature mortality rates to be overestimated, a phenomenon that is not mirrored in the NHW population. This issue is largely attenuated in DP22, with an average percent bias of 0.162% in the racialized inequity parameter estimate. In MA, the poverty coefficient is generally estimated with little bias for all denominator data sources, although DP20 yields slightly more bias than the other denominators.

**Fig. 4.**
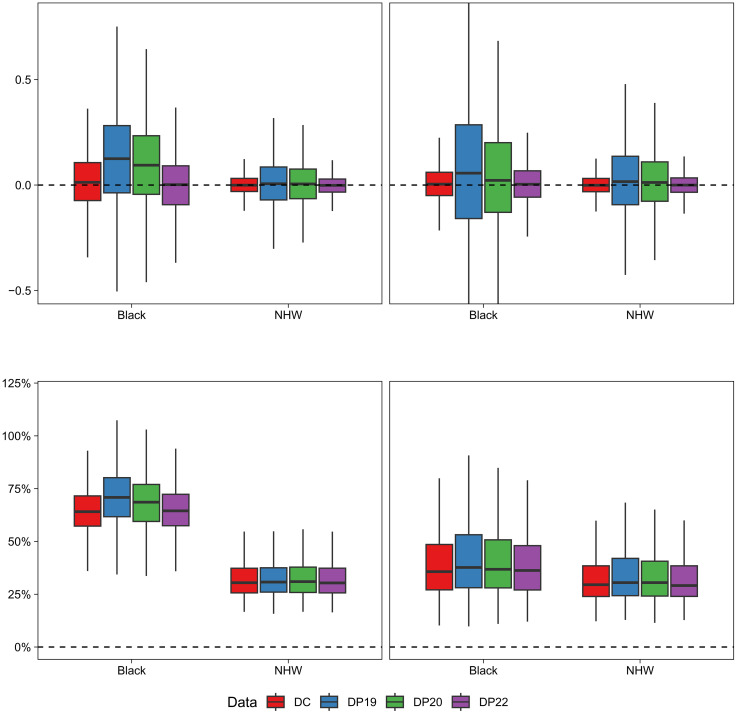
Simulation results: Bias and MAPE in model-estimated census tract–level race-stratified SMRs using different denominator data sources. Boxplots represent the distribution of bias (**top row**) and MAPE (**bottom row**) in the across-simulation average SMR estimates for each census tract, with simulated data mimicking patterns of premature mortality in Massachusetts (**left column**) and Georgia (**right column**) and models fit using each of the four denominator data sources: the 2010 DC and the U.S. Census Bureau DAS demonstration products released in 2019 (DP19), 2020 (DP20), and 2022 (DP22). Data were generated using DC as the true denominator data.

To further investigate whether patterns in model-smoothed area-specific disease/mortality risk estimates are preserved when using the DAS-protected denominators, we also evaluate the bias and mean absolute percent error (MAPE) in the model-estimated SMRs using each denominator data source (see the “Model assessment metrics” section for details on how these were calculated). Figure 4 shows boxplots of the bias and MAPE in the model-estimated SMRs using each denominator data source for each state’s simulation study (numeric summaries are provided in tables S6 and S7). Note that, in these plots, the data represented are the census tract– and racialized group–specific bias/MAPEs, averaged across simulations (see the “Model assessment metrics” section), as opposed to Fig. 3 where the data are the difference between an estimate and the truth for each individual simulation. When using the true DC denominators in the models, estimated SMRs are unbiased on average for both racialized groups and both states. However, the MAPEs are much larger for the Black SMRs compared to the NHW SMRs. This is especially pronounced in MA, where the MAPEs for the Black population tend to be more than double those for the NHW population. This indicates that even correctly specified models, in the absence of error in the denominators, struggle more to estimate Black (compared to NHW) census tract–level SMRs. The likely cause of this phenomenon is the smaller population sizes and more unstable rates for the Black population in most census tracts, which also explains why the impacts are more profound in MA.

The use of DP19 and DP20 denominators exacerbates this disparity in SMR estimation accuracy in MA. With these denominators, SMR estimates for the NHW population remain unbiased on average, but for the Black population, even the average of the SMR estimates demonstrates a substantial upward bias (Fig. 4). This is further illustrated in table S8, which provides the percent of SMRs biased upward for both racialized groups for each of the four denominator data sources. This is, again, the result of the systematic underestimation of the DAS-protected denominators for Black populations in MA (Fig. 2). There is also a larger DC versus DP19/DP20 differential in the distribution of MAPEs for the Black population (relative to NHW), indicating that the use of these DAS variants worsens the (already poorer) model performance for Black populations more than for NHW populations. However, using the DP22 denominators, the distributions of SMR biases and MAPEs for MA are virtually indistinguishable from those observed when using the DC denominators, indicating that using the newest DAS variant for SMR estimation gives results with nearly identical accuracy to the DC. For GA, a similar but less pronounced pattern emerges, with DP19 and DP20 yielding SMRs with slightly higher bias and MAPE than DC, with a larger differential for the Black population. Again, the performance of the DP22 denominators is almost identical to that of the DC denominators.

**Fig. 5.**
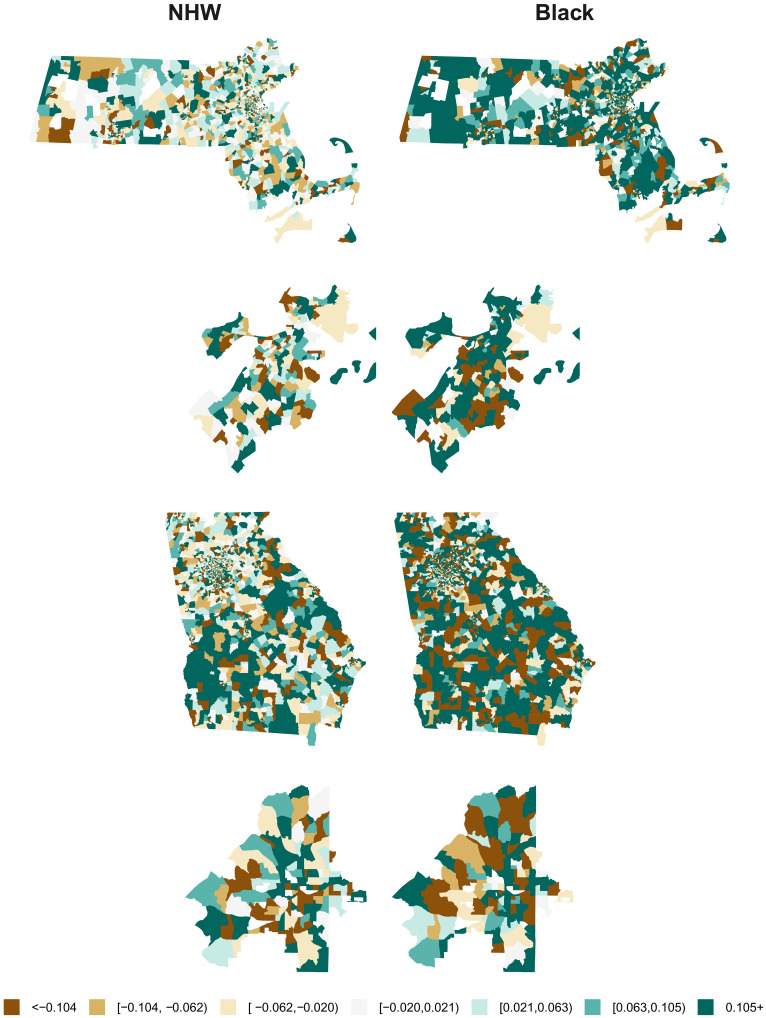
Simulation results: Maps of bias in model-estimated census tract–level SMRs by racialized group using 2020 demonstration product denominators. Maps show bias in the SMR estimates, averaged across simulations, for the NHW (**left column**) and Black (**right column**) populations from models fit using denominator data from the U.S. Census Bureau DAS demonstration product released in 2020. Simulated data were generated to mimic patterns of premature mortality in Massachusetts and Georgia and used 2010 DC denominators as the true denominators. Maps show Massachusetts (**first row**), Boston (**second row**), Georgia (**third row**), and Atlanta **(fourth row**).

**Fig. 6.**
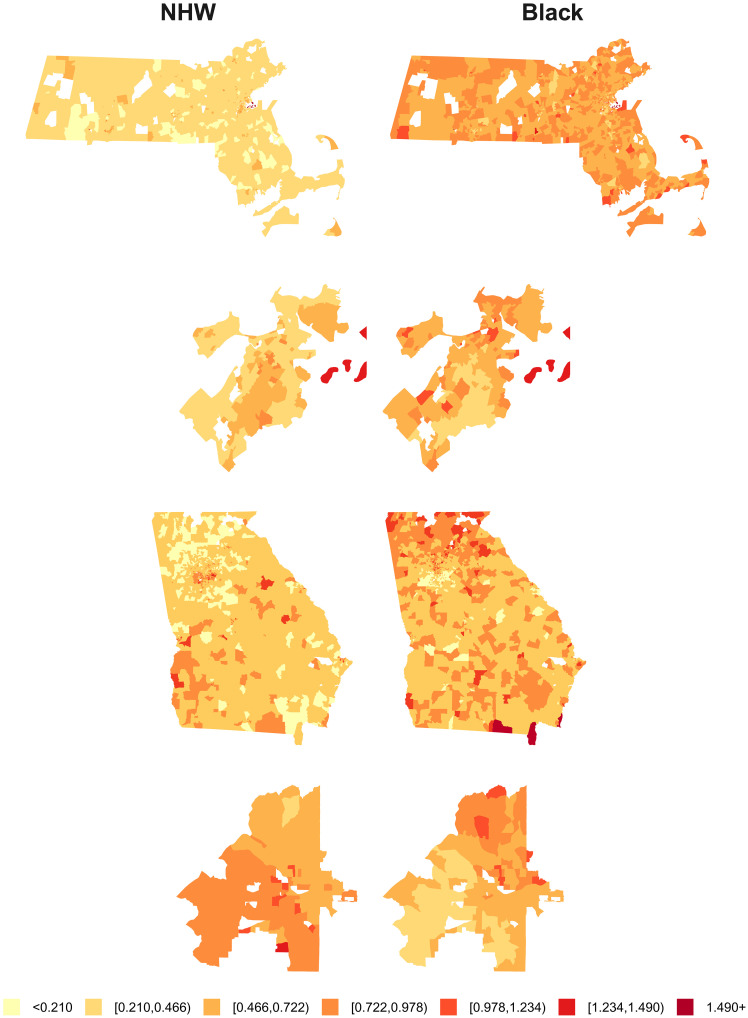
Simulation results: Maps of MAPE in model-estimated census tract–level SMRs by racialized group using 2020 demonstration product denominators. Maps show MAPE in the SMR estimates for the NHW (**left column**) and Black (**right column**) populations from models fit using denominator data from the U.S. Census Bureau DAS demonstration product released in 2020. Simulated data were generated to mimic patterns of premature mortality in Massachusetts and Georgia and used 2010 DC denominators as the true denominators. Maps show Massachusetts (**first row**), Boston (**second row**), Georgia (**third row**), and Atlanta (**fourth row**).

The biases and MAPEs for the model-estimated SMRs for each census tract and racialized group using the DP20 denominators are mapped in Figs. 5 and 6 (the patterns for DP19 are similar and the maps for DP22 are shown in figs. S4 and S5). In Fig. 6, the patterns in the MAPE maps for the Black population are nearly inverted from those in the NHW maps, and from those in the percent Black map in Fig. 1. Census tracts with larger populations for a given racialized group tend to have smaller SMR errors for that group, and vice versa. This corroborates the findings of previous studies that have suggested that more severe distortions may occur when using DAS denominators to characterize health-related patterns in smaller population groups (*1*, *26*). Our findings, including the presence of more blue/green hues in the maps of bias in the Black SMRs for MA (Fig. 5), provide further insight that the use of DAS denominators using smaller privacy-loss budgets may tend to favor overestimation for Black premature mortality rates but not for NHW rates in areas with small, concentrated Black populations (as described in Fig. 4). However, as we have consistently reported throughout this section, these distortions are largely eliminated by increasing the privacy-loss budget to the values applied in DP22.

The setup and results of a secondary simulation study emulating a rare disease/mortality outcome are described in detail in text S1. Briefly, we found that the DAS denominators had less pronounced impacts on the model results in the rare outcome setting, likely due to the greater instability/variability in all models, including those using the true DC denominators, which may eclipse the influence of denominator errors on the models.

## DISCUSSION

Here, we explored the potential impact of the U.S. Census Bureau’s proposed 2020 DC DAS procedures, including the use of differential privacy, taking three additional steps beyond those in the handful of investigations focusing on this topic in relation to health and health inequities, by (i) modeling small-area disease/mortality rates for the purposes of identifying health inequities, (ii) including the newest released demonstration product at the time of writing that incorporates the recent Census Bureau refinements to the DAS, and (iii) conducting simulation analyses to formally quantify the biases introduced by the DAS in mortality rate estimation for Black and NHW populations separately. Using three DAS-protected 2010 census demonstration products released by the Census Bureau, we conducted a simulation study and an analysis of real small-area premature mortality data from two demographically distinct states to investigate how the DAS procedures affected racialized group and economic inequity estimates. Our results provide evidence that recent changes to the DAS procedures made by the Census Bureau in response to stakeholder feedback, featured in the August 2022 demonstration product, brought about substantial improvements in accuracy of the DAS-protected denominators relative to older variants of the DAS, with respect to the accuracy metrics considered here. This increased accuracy translates to sizeable decreases in biases in disease mapping and inequity studies using these denominators, particularly when interest lies in disease/mortality rate estimation and quantification of inequities for small population groups. We observed that biases in racialized and economic inequity parameter estimates and model-based SMR estimates from models using the DP22 denominators were virtually indistinguishable from those obtained when using the original DC data. If the Census Bureau implements the 2020 DAS with settings similar to those used in the August 2022 demonstration product, our findings suggest that there is likely to be little, if any, DAS-induced degradation of accuracy in small-area disease mapping and health inequity studies using these data as population denominators (relative to the accuracy obtained using census data under the previously used disclosure avoidance procedures).

Our work demonstrates (i) the profound implications of the U.S. Census Bureau’s choice of privacy-loss budget for the accuracy of future small-area disease mapping and health inequity studies and (ii) the extent to which a small privacy-loss budget, combined with modeling approaches that neglect the DAS-induced error in denominators for small groups, can distort characterizations of population health. In both the MA and GA case studies, we found that high-level patterns in inequities by racialized group and socioeconomic status were preserved when using any of the demonstration products. However, when using older demonstration products (DP19 and DP20) that applied differential privacy with a lower privacy-loss budget, the errors induced in mortality rate estimation were considerably larger for Black than NHW populations. This phenomenon was especially pronounced in MA, which has a small Black population. In particular, in the example examined here, these older DAS variants led to systematic underestimation of denominators and, therefore, overestimation of premature mortality rates for Black populations in MA, which was not observed for NHW populations. These relatively larger DAS-induced errors in estimated small-area SMRs for Black populations relative to NHW populations compound the already worse model performance for Black populations due to small counts and rate instability.

Our findings regarding the older DAS variants generally agree with some previous studies, which have reported that DAS-protected denominators are more problematic for estimation of rates in smaller racialized groups (*1*, *26*). Such mischaracterizations could have real implications for public health practitioners, who rely on these metrics for identification of an intervention on high-risk groups. For instance, these distortions may lead policymakers to miss opportunities to improve public health in some small populations and/or small areas, which due to myriad social and economic factors are often the groups with the highest health risks. However, encouraging evidence from our investigation of the most recent demonstration product (DP22) suggests that such issues can be largely ameliorated, in this context, by the choice of a larger privacy-loss budget in the differential privacy implementation. Thus, the use of DAS procedures similar to those in DP22 may strike a compromise between preservation of privacy and the ability to accurately characterize and advance the health of even small populations and areas.

To our knowledge, our study is so far the first study to compare different DAS-protected 2010 census demonstration products’ impacts for small-area disease modeling and inequity studies, and it is the first health-focused study to investigate the demonstration product newly released in August 2022. The primary weakness of our study is that we only investigate DAS impacts in two states, MA and GA, and on health inequity estimates for the two largest racialized groups in these states, Black and NHW. The DAS-attributable errors uncovered here may be further exacerbated for other smaller groups, such as Native Americans and Asian Americans/Pacific Islanders.

Another limitation of our study is the use of the public release 2010 DC data as a baseline. As described in the “Population count data” section, these data are also potentially affected by errors from a vintage privacy-preserving procedure known as data swapping. Even more fundamentally, the DC population counts are known to exhibit systematic biases due to data collection challenges, with a particular tendency to underrepresent non-white individuals, which is troublesome for health inequity studies (*35*). However, these data represent the current gold standard and have been used as a baseline for accuracy assessments of the demonstration products in many other studies (*1*, *13*, *14*, *27*) and in the U.S. Census Bureau’s internal assessments (*7*, *28*). Moreover, our findings that the DAS demonstration products that impose a higher privacy-loss budget (and therefore have higher accuracy) are most similar to the 2010 DC data provide further validation of the use of the DC as a baseline for accuracy assessment. As the implementation of differential privacy to preserve privacy in publicly released health and social science data accelerates, the development of statistical methods to adapt standard disease mapping models to differential privacy-injected noise in variables is critical to reduce systematic errors that can bias health inequity estimates. Future work should also investigate how the Census Bureau’s 2020 DAS affects health inequity studies using smaller populations, such as Native Americans and Asian Americans/Pacific Islanders.

## MATERIALS AND METHODS

### Differences in DAS demonstration products

In the differential privacy algorithm, one of the key quantities controlling the accuracy privacy-loss trade-off is the privacy-loss budget parameter, ε, representing the spectrum between perfect privacy/no accuracy (ε → 0), to perfect accuracy/no privacy (ε → ∞). Formally, ε is a probabilistic bound on disclosure risk on the log scale. An increase in ε will generally improve the quality of the data product (*36*) [although accuracy can still decline with respect to utility metrics not accounted for in the differential privacy algorithm (*37*)]. While other parameters also influence the privacy/accuracy trade-off, those are less emphasized by the Census Bureau in their DAS documentation and so for simplicity are omitted from our discussion here. The DP19 and DP20 demonstration products use the same value of ε (ε = 6.0), and thereby identical implementations of differential privacy.

The difference between DP19 and DP20 lies only in the postprocessing procedures, which are operations involving how the DAS algorithm converts the formally private noisy tabulations taken from the confidential data into the non-negative integer counts that will be published. Critically, the postprocessing ensures that the data obey certain practical constraints, e.g., that the sum of counts from small areas nested within a large area is equal to the count for the large area. The algorithm used in DP19 conducted the postprocessing of all of the statistics for a particular geographic level at the same time, resulting in distortions when there were large quantities of statistics with zeros or very small values processed at the same time. To address and mitigate this issue, the algorithm used in DP20 conducts the postprocessing in a series of passes through all the geographic levels (national level, state level, etc.) (*38*). Specifically, the first pass processed total population counts, and the second pass processed statistics necessary to inform redistricting. The third pass processed core statistics stratified by age/sex/racialized group, and the final pass processed all remaining counts. In this version of the algorithm, output from each pass was constrained to agree with the counts from prior passes.

DP22, on the other hand, incorporates modifications to the differential privacy algorithm parameters in response to stakeholder feedback that greater accuracy was needed. Specifically, the Census Bureau tuned the privacy-loss budget applied to different sets of tabulations (*39*). The global privacy-loss budget for DP22 is ε = 46.24 (*40*), which is substantially higher than the analogous privacy-loss budget for DP19 and DP20, yielding counts with lower privacy/higher accuracy. This change substantially weakened the privacy guarantee, but it allowed the Census Bureau to meet accuracy targets identified by data user communities. Moreover, DP22 applies the same multipass postprocessing procedures as DP20 but incorporates additional geographic entities into this postprocessing (*40*). DP22 is the most recently released demonstration product and reflects the most up-to-date refinements of the DAS procedures at this time.

### Data collection and processing

#### Population count data

For the original DC data, we use the tidycensus R package (*41*) (for convenience) to extract census tract population counts stratified by age and census-defined “racial” and “ethnic” categories [which we refer to as racialized groups (*42*), since these categories are socially constructed]. In our study, we consider these to be baseline population counts. For the three demonstration data products, we obtain population counts stratified by age and racialized group from the IPUMS (Integrated Public Use Microdata Series) National Historical Geographic Information System website (*43*).

#### Outcome data

The MA premature mortality counts are for the year 2010 and were collected from the MA Department of Public Health (*44*). These data have been described previously [see Krieger *et al.* (*27*) for more details]. The GA premature mortality counts are for the period 2008–2012 and were obtained from the GA Department of Public Health (*45*). Because the only human data relied upon in this study are death records, this work was determined to be not human subjects research by the Institutional Review Board of the Harvard T.H. Chan School of Public Health.

#### Age standardization

To perform indirect age standardization and create expected premature mortality counts to be used as denominators based on the DC, DP19, DP20, and DP22 population counts, we use the ageadjust.indirect() function from the epitools R package (*46*). Age standardization adjusts for differences in age distribution to mitigate possible confounding effects on inequity analyses arising from differing age distributions across groups. We conduct age standardization, based on empirical MA and GA statewide age group–specific premature mortality rates, separately for each census tract and racialized group, to get expected premature mortality counts. Throughout the article, we consistently use 1-year expected counts for the year 2010, except in the models fit to the real GA premature mortality data, where we use 5-year expected counts (created by multiplying the 1-year expected counts by 5) to align with the 5-year premature mortality counts.

### Real data analysis models

Throughout our analyses, all computations and modeling are conducted separately, but analogously, for the two states. For each state, the four sets of SMRs are modeled separately, using a multilevel spatial Poisson regression model (*47*). The model specifications were selected to broadly represent a class of models commonly used in public health inequities research (*48*–*56*). Let *i* = 1, …, *N* index census tracts and *j* = 0,1 index racialized group (0 for NHW and 1 for Black) so that *Y_ij_* is the premature mortality count in racialized group *j* within census tract *i*. I(Black)*_ij_* is a binary indicator of Black racialized group, and PropPov*_i_* is the proportion in poverty in census tract *i* (centered and scaled). Let *P_ij_* be the census tract– and racialized group–specific expected number of premature mortalities computed using any of the four population count data sources. We assume *Y_ij_* ∼ Poisson (*λ_ij_*) and fit the following model using each of the four variants of *P_ij_* formed from DC, DP19, DP20, and DP22

$$\log\;(\lambda_{ij})=\beta_{0}+\beta_{1}I{\left(\mathrm{Black}\right)}_{ij}+\beta_{2}\mathrm{PropPo}v_{i}+\theta_{i}+\phi_{ij}+\log\;(P_{ij})$$ (1)

where θ*_i_* is a census tract–specific random effect with a conditionally autoregressive spatial covariance structure (*57*) and ϕ*_ij_* is an unstructured census tract– and racialized group–specific error term that models overdispersion in the disease count. To clarify, θ*_i_* is spatial random intercept unique to census tracts but shared by racialized groups within a census tract, while ϕ*_ij_* are both census tract– and racialized group–specific. Models are fit by a Bayesian approach implemented in the CARBayes package in R (*58*). For each state and each of the four denominator data sources, mortality rate ratio estimates based on the exponentiated posterior means of the coefficients and 95% credible intervals are reported and compared.

### Simulation study details

#### Data generating process

Using the 2010 DC expected counts for each census tract and racialized group, we simulate the outcomes. Formally, premature mortality counts, *Y*, are generated following the model form in Eq. 1, plugging in the real DC-based expected counts for *P* and using the real census tract–level PropPov variable. The coefficient parameter values used in all simulations are β_0_ = 0, β_1_ = 0.4, and β_2_ = 0.01.

The conditionally autoregressive spatial effect θ*_i_* is generated as $\theta_{i}|\theta_{-i}∼\mathrm{Normal}\;(0.2w_{i+}^{-1}\sum_{g}w_{ig}y_{g},w_{i+}^{-1})$, where *w_ig_* is the (*i*, *g*)^th^ element of a census tract adjacency matrix *W*, and *w*_*i*+_ is the sum of the elements in the *i*^th^ row of *W*. ϕ*_ij_* ∼ Normal (0,0.25) is an unstructured random effect. The hyperparameter values in the distributions of θ*_i_* and ϕ*_ij_* were selected to generate data with moderate spatial correlation and an outcome distribution mimicking the empirical distribution of census tract premature mortality rates in our case study data.

We simulate 100 datasets from this model, and for each simulated dataset, we fit four models to it—one plugging in each set of expected counts (DC, DP19, DP20, and DP22) as the denominator. Aside from possible error in the denominators, the fitted models are otherwise correctly specified to allow us to isolate potential biases due to DAS-induced error in the denominators.

#### Model assessment metrics

For a given simulated dataset (indexed by *k* = 1, …,100) and denominator data source (*P_ij_*), we estimate the model-based SMRs as

$${\hat{\mathrm{SMR}}}_{ijk}=\frac{{\hat{Y}}_{ijk}}{P_{ij}}$$ (2)

where ${\hat{Y}}_{ijk}$ is the predicted value of *Y_ij_* from the model fit to simulated dataset *k* using the given denominator data source. We then compute the bias and MAPE for each census tract and racialized group’s SMR estimate for each denominator data source, i.e.,

$${\mathrm{MAPE}}_{ij}=\frac{1}{100}\sum_{k=1}^{100}|\frac{{\hat{\mathrm{SMR}}}_{ijk}-(\lambda_{ijk}/P_{ij})}{\left(\lambda_{ijk}/P_{ij}\right)}|$$ (3)

$${\mathrm{Bias}}_{ij}=\frac{1}{100}\sum_{k=1}^{100}({\hat{\mathrm{SMR}}}_{ijk}-(\lambda_{ijk}/P_{ij}))$$ (4)
