# Supplementary material for: Impacts of census differential privacy for small-area disease mapping to monitor health inequities
Source: Sci Adv. 2023 Aug 18;9(33):eade8888. doi: 10.1126/sciadv.ade8888 (PMC10438951; doi:10.1126/sciadv.ade8888)

Science Advances
AAAS

Supplementary Materials for

Impacts of census differential privacy for small-area disease mapping to monitor health inequities

Yanran Li *et al.*

Corresponding author: Rachel C. Nethery, rnethery@hsph.harvard.edu

Sci. Adv. **9**, eade8888 (2023)
DOI: 10.1126/sciadv.ade8888

This PDF file includes:

Text S1
Figs. S1 to S5
Tables S1 to S8

S Supplementary Materials: Impacts of Census Differential Privacy for Small-Area Disease Mapping to Monitor Health Inequities

S.1 Secondary simulation study

We conducted a secondary simulation study to evaluate the potential DAS impacts in a rare disease/mortality outcome setting in Massachusetts (MA). To obtain synthetic data mimicking a rarer outcome, we used the same data generating mechanism as described in Section 4.4.1 of the main manuscript, only changing the intercept value to $\beta_0 = -0.5$. We used the same model fitting and assessment procedures for each simulated dataset as described in the main text.

Figure S.1 shows boxplots of the percent bias in the estimated model coefficients across the simulations, using each of the four denominator data sources. The coefficient biases from models using all denominator data sources, including the true DC denominators, demonstrate more spread/variability in the rare outcome setting than in the primary simulation study in the main text (Figure 3). This is likely due to greater instability in all models due to the rare and zero-heavy outcome. However, the on-average biases occurring in the racialized group coefficient parameter when using the DP19/DP20 denominators identified in the primary simulation study are not as evident here. One possible explanation for this is that the increased variability/instability in the coefficient estimates induced by the rare outcomes obscures the on-average biases due to denominator errors.

Bias and mean absolute percent errors (MAPEs) in the model-estimated census tract SMRs are shown in Figure S.2. These results display similar patterns to the analogous results from the primary simulation study (in Figure 4 of the main manuscript), although consistent with our findings for the coefficient biases, the errors in SMRs induced by the DP19/DP20 denominators are less pronounced. This is likely because both the true and model-estimated event rates (λ_{ij}) are near the lower bound of zero for rare outcomes. Near this lower bound, there may be a smaller margin for denominator errors to propagate into model estimation errors.

Figure S.1: Simulation results: percent bias in estimated inequity model coefficients (with decreased β_0 value) using different denominator data sources. Boxplots represent the distribution of bias in estimates of the Black vs non-Hispanic White (NHW) (first column) and Percent Poverty (second column) coefficients over 100 simulations, with simulated data mimicking patterns of premature mortality in Massachusetts and models fit using each of the four denominator data sources: the 2010 decennial census (DC) and the US Census Bureau Disclosure Avoidance System demonstration products released in 2019 (DP19), 2020 (DP20), and 2022 (DP22). Data were generated using DC as the true denominator data.

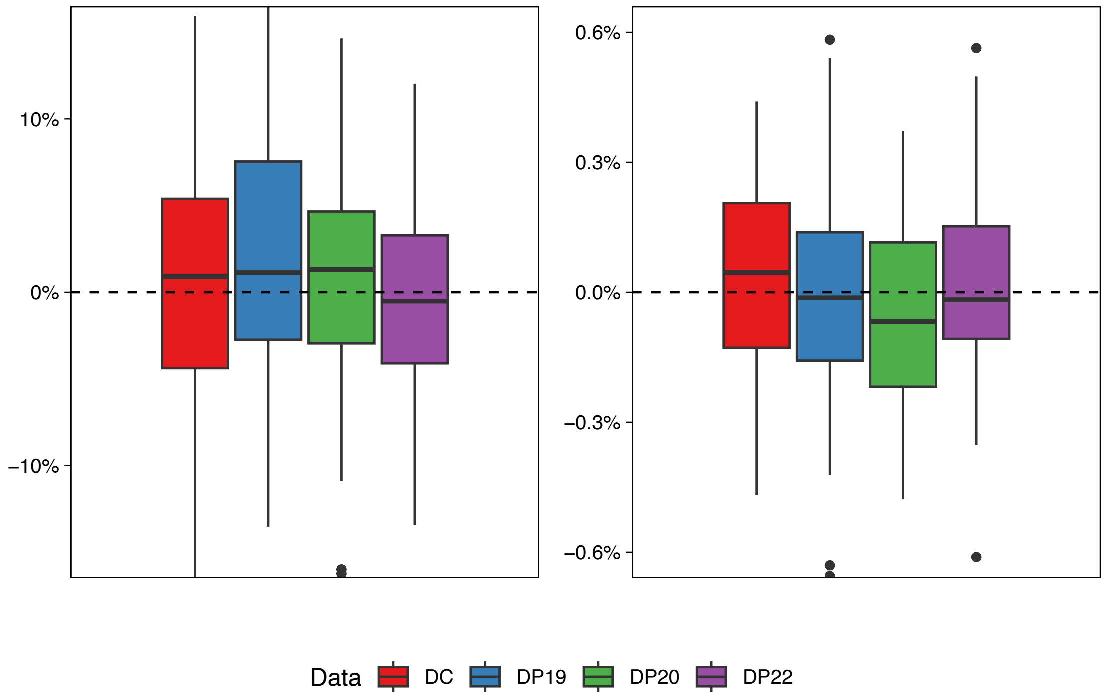

Figure S.2: Simulation results: bias and mean absolute percent error (MAPE) in model-estimated census tract-level race-stratified standardized mortality ratios (SMR) using different denominator data sources (with decreased β_0 value). Boxplots represent the distribution of bias (left column) and MAPE (right column) in the across-simulation average SMR estimates for each census tract, with simulated data mimicking patterns of premature mortality in Massachusetts and models fit using each of the four denominator data sources: the 2010 decennial census (DC) and the US Census Bureau Disclosure Avoidance System demonstration products released in 2019 (DP19), 2020 (DP20), and 2022 (DP22). Data were generated using DC as the true denominator data.

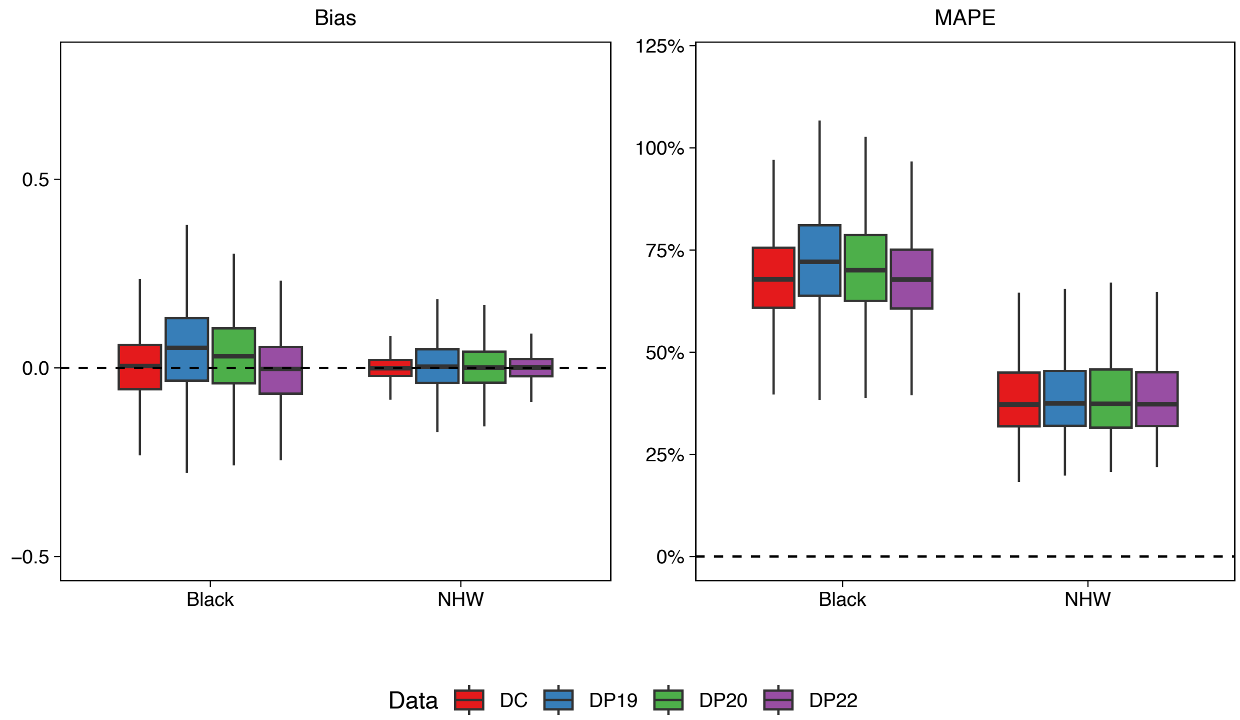

S.2 Supplementary tables and figures

Table S.1: Summary statistics (Min, Max, 25th, 50th, and 75th percentiles) of the difference in census tract expected premature mortality counts for 2010 created from the US Census Bureau Disclosure Avoidance System demonstration products released in 2019 (DP19), 2020 (DP20), and 2022 (DP22), relative to original 2010 decennial census-based expected counts, for Black and non-Hispanic white populations in Massachusetts and Georgia.

	Data Sources	Racialized Group	Min	25th	50th	75th	Max
Massachusetts	DP19	NHW	-2.6348	-0.3293	-0.0026	0.3548	1.7335
		Black	-0.9207	-0.0515	-0.0163	0.0371	1.1627
	DP20	NHW	-1.8218	-0.3212	-0.0049	0.3066	1.6439
		Black	-0.4807	-0.0404	-0.0093	0.0245	0.6865
	DP22	NHW	-0.1713	-0.0327	0.0003	0.0345	0.1615
		Black	-0.1296	-0.0141	-0.0015	0.0138	0.1123
Georgia	DP19	NHW	-2.9548	-0.4979	-0.0308	0.4685	3.4053
		Black	-3.2302	-0.3005	-0.0393	0.2698	2.6863
	DP20	NHW	-2.3173	-0.3956	-0.0184	0.3593	2.6525
		Black	-2.2789	-0.2213	-0.0163	0.2208	2.3190
	DP22	NHW	-0.4066	-0.0563	-0.0005	0.0550	0.3055
		Black	-0.2878	-0.0495	-0.0008	0.0489	0.3683

Table S.2: Percent of census tract expected premature mortality counts for 2010 created from the US Census Bureau Disclosure Avoidance System demonstration products released in 2019 (DP19), 2020 (DP20), and 2022 (DP22), that are under-estimated relative to original 2010 decennial census-based expected counts, for Black and non-Hispanic white (NHW) populations in Massachusetts and Georgia.

		DP19	DP20	DP22
Massachusetts	NHW	50.3084	50.3770	49.9657
	Black	62.7645	59.3794	52.8209
Georgia	NHW	52.2587	51.5914	50.2053
	Black	55.3783	52.5476	50.4889

Table S.3: Percent of 2010 census tract-level racialized group-specific expected counts that are 0 and the percent that are less than 5 for each of the four denominator data sources: the 2010 decennial census (DC) and the US Census Bureau Disclosure Avoidance System demonstration products released in 2019 (DP19), 2020 (DP20), and 2022 (DP22).

		NHW				Black			
	Data Sources	DC	DP19	DP20	DP22	DC	DP19	DP20	DP22
Massachusetts	0 expected counts (%)	0.00	0.00	0.07	0.00	0.14	1.64	1.37	0.07
	<5 expected counts (%)	39.32	38.90	39.32	39.18	100.00	100.00	100.00	100.00
Georgia	0 expected counts (%)	0.00	0.26	0.10	0.00	0.10	0.20	0.41	0.05
	<5 expected counts (%)	43.43	44.55	44.40	43.53	80.51	80.46	80.66	80.15

Table S.4: Simulation results: average biases in estimated inequity model coefficients using different denominator data sources. Average bias in estimates of the Black vs non-Hispanic White and Percent Poverty coefficients over 100 simulations, with simulated data mimicking patterns of premature mortality in Massachusetts and Georgia and models fit using each of the four denominator data sources: the 2010 decennial census (DC) and the US Census Bureau Disclosure Avoidance System demonstration products released in 2019 (DP19), 2020 (DP20), and 2022 (DP22). Data were generated using DC as the true denominator data.

		Data Sources			
		DC	DP19	DP20	DP22
Massachusetts	Intercept	-0.0006	-0.0046	0.0114	-0.0025
	Racialized group	0.0016	0.0386	0.0258	0.0000
	Poverty	0.0001	0.0001	-0.0008	0.0002
Georgia	Intercept	-0.0042	0.0048	0.0030	-0.0016
	Racialized group	0.0032	0.0051	-0.0022	0.0013
	Poverty	0.0002	0.0003	0.0001	0.0001

Table S.5: Simulation study results: 95% credible interval coverage percentage (%) for the coefficient parameters in simulations mimicking real data from Massachusetts and Georgia and models fit using each of the four denominator data sources: the 2010 decennial census (DC) and the US Census Bureau Disclosure Avoidance System demonstration products released in 2019 (DP19), 2020 (DP20), and 2022 (DP22). Data were generated using DC as the true denominator data. β_0 is the intercept, β_1 is the coefficient parameter for the Black vs non-Hispanic white indicator, and β_2 is the coefficient parameter for the percent in poverty variable.

		Data Sources			
		DC	DP19	DP20	DP22
Massachusetts	β_0	95	91	83	91
	β_1	98	89	94	97
	β_2	98	95	93	98
Georgia	β_0	93	97	92	89
	β_1	97	97	94	92
	β_2	92	97	95	94

Table S.6: Simulation results: average bias in model-estimated census tract-level race-stratified standardized mortality ratios (SMR) using different denominator data sources. Values represent averages of the differences in true census tract SMRs and across-simulation average SMR estimates, with simulated data mimicking patterns of premature mortality in Massachusetts and Georgia and models fit using each of the four denominator data sources: the 2010 decennial census (DC) and the US Census Bureau Disclosure Avoidance System demonstration products released in 2019 (DP19), 2020 (DP20), and 2022 (DP22). Data were generated using DC as the true denominator data.

		Data Sources			
		DC	DP19	DP20	DP22
Massachusetts	NHW	0.0006	0.0164	0.0104	-0.0032
	Black	0.0049	0.1281	0.0899	-0.0058
Georgia	NHW	-0.0013	0.0445	0.0255	-0.0008
	Black	0.0028	0.0852	0.0419	0.0035

Table S.7: Simulation results: average of mean absolute percent errors (MAPE) in model-estimated census tract-level race-stratified standardized mortality ratios (SMR) using different denominator data sources. Values represent averages of the MAPEs for the census tract-level across-simulation average SMR estimates, with simulated data mimicking patterns of premature mortality in Massachusetts and Georgia and models fit using each of the four denominator data sources: the 2010 decennial census (DC) and the US Census Bureau Disclosure Avoidance System demonstration products released in 2019 (DP19), 2020 (DP20), and 2022 (DP22). Data were generated using DC as the true denominator data.

		Data Sources			
		DC	DP19	DP20	DP22
Massachusetts	NHW	0.3313	0.3412	0.3391	0.3320
	Black	0.6447	0.7123	0.6886	0.6487
Georgia	NHW	0.3314	0.3583	0.3455	0.3307
	Black	0.3853	0.4222	0.4034	0.3863

Table S.8: Simulation results: percent of model-estimated census tract-level race-stratified standardized mortality ratios (SMR) that are biased upwards using different denominator data sources. Values represent the percent of census tract-level across-simulation average SMR estimates that are lower than the corresponding true SMR, with simulated data mimicking patterns of premature mortality in Massachusetts and Georgia and models fit using each of the four denominator data sources: the 2010 decennial census (DC) and the US Census Bureau Disclosure Avoidance System demonstration products released in 2019 (DP19), 2020 (DP20), and 2022 (DP22). Data were generated using DC as the true denominator data.

		Data Sources			
		DC	DP19	DP20	DP22
Massachusetts	NHW	49.3489	52.0905	51.8849	48.9376
	Black	54.0197	70.2398	68.3357	50.4937
Georgia	NHW	48.9733	54.3634	53.0801	49.7433
	Black	51.7241	57.2311	53.1652	51.7756

Figure S.3: Scatterplots of 2010 decennial census (DC) expected premature mortality counts (x-axis) versus 2010 demonstration product expected counts (y-axis) for Massachusetts (first row) and Georgia (second row) census tracts for the non-Hispanic White (NHW) (left column) and Black (right column) populations.

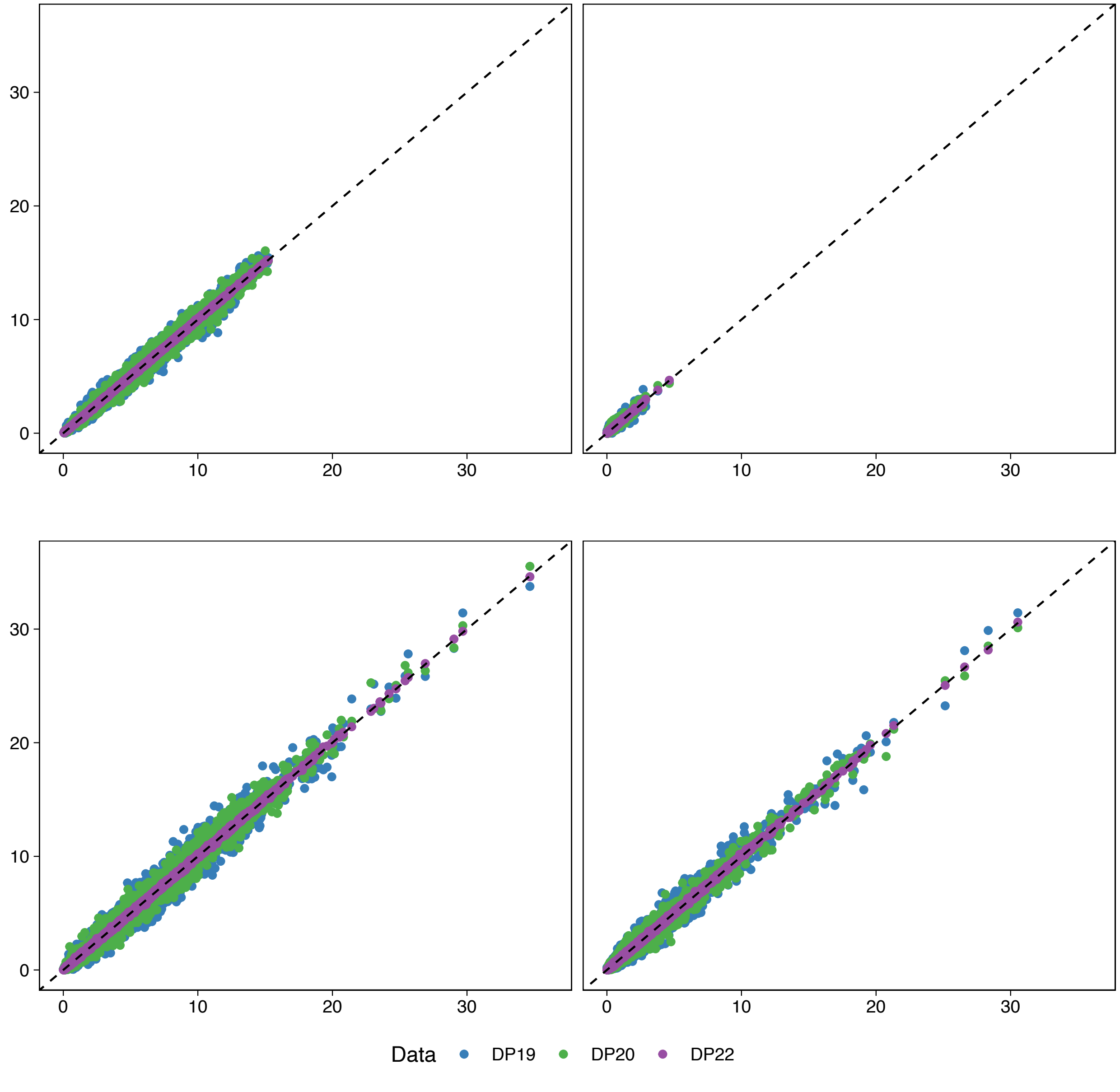

Figure S.4: Simulation results: maps of bias in model-estimated census tract-level standardized mortality ratios (averaged across simulations) for the non-Hispanic White (NHW) (left column) and Black (right column) populations from models fit using denominator data from the US Census Bureau Disclosure Avoidance System demonstration product released in 2022. Simulated data were generated to mimic patterns of premature mortality in Massachusetts and Georgia and used 2010 decennial census denominators as the true denominators. Maps show Massachusetts (first row), Boston (second row), Georgia (third row) and Atlanta (fourth row).

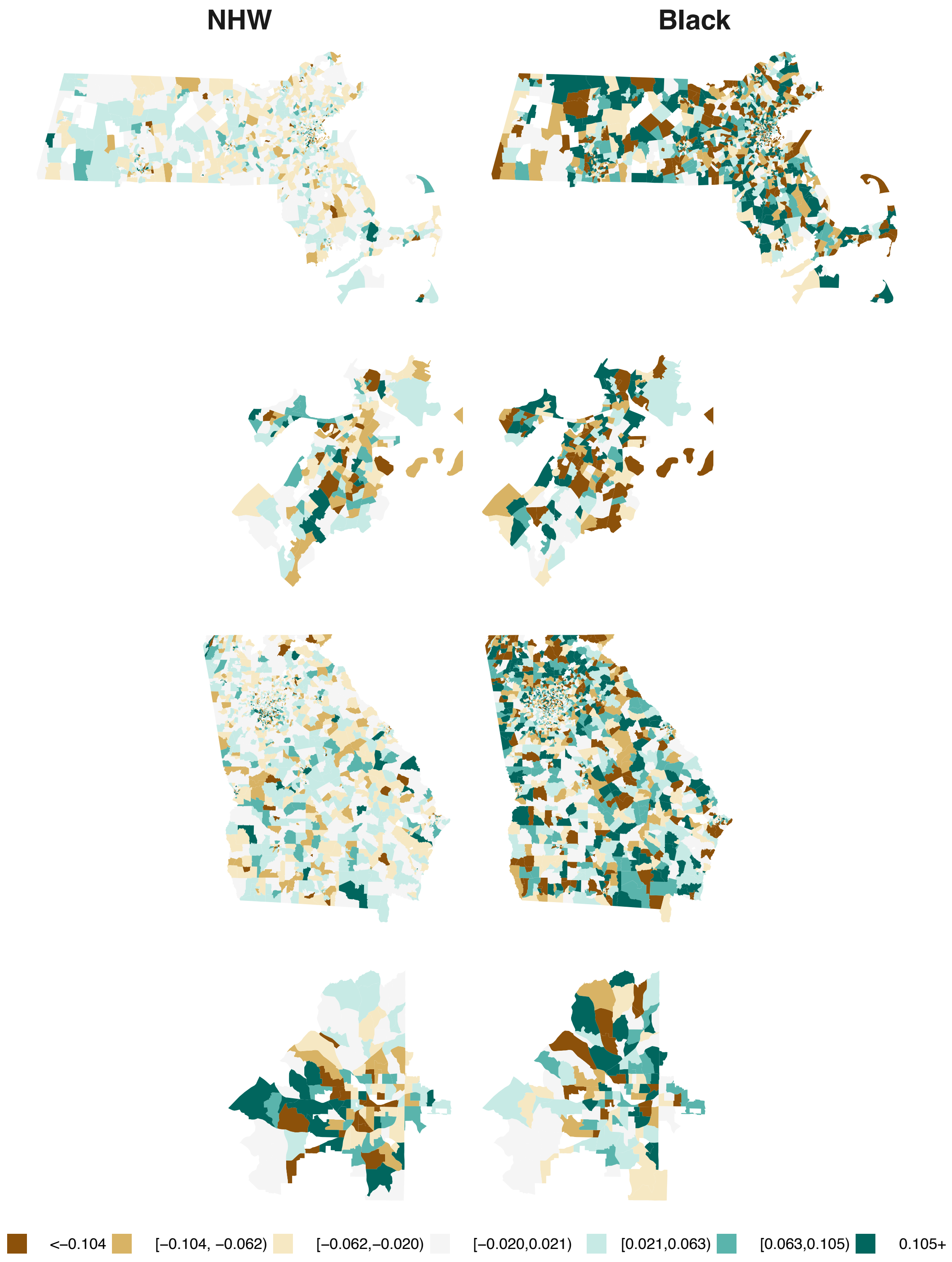

Figure S.5: Simulation results: maps of mean absolute percent error in model-estimated census tract-level standardized mortality ratios (averaged across simulations) for the non-Hispanic White (NHW) (left column) and Black (right column) populations from models fit using denominator data from the US Census Bureau Disclosure Avoidance System demonstration product released in 2022. Simulated data were generated to mimic patterns of premature mortality in Massachusetts and Georgia and used 2010 decennial census denominators as the true denominators. Maps show Massachusetts (first row), Boston (second row), Georgia (third row) and Atlanta (fourth row).

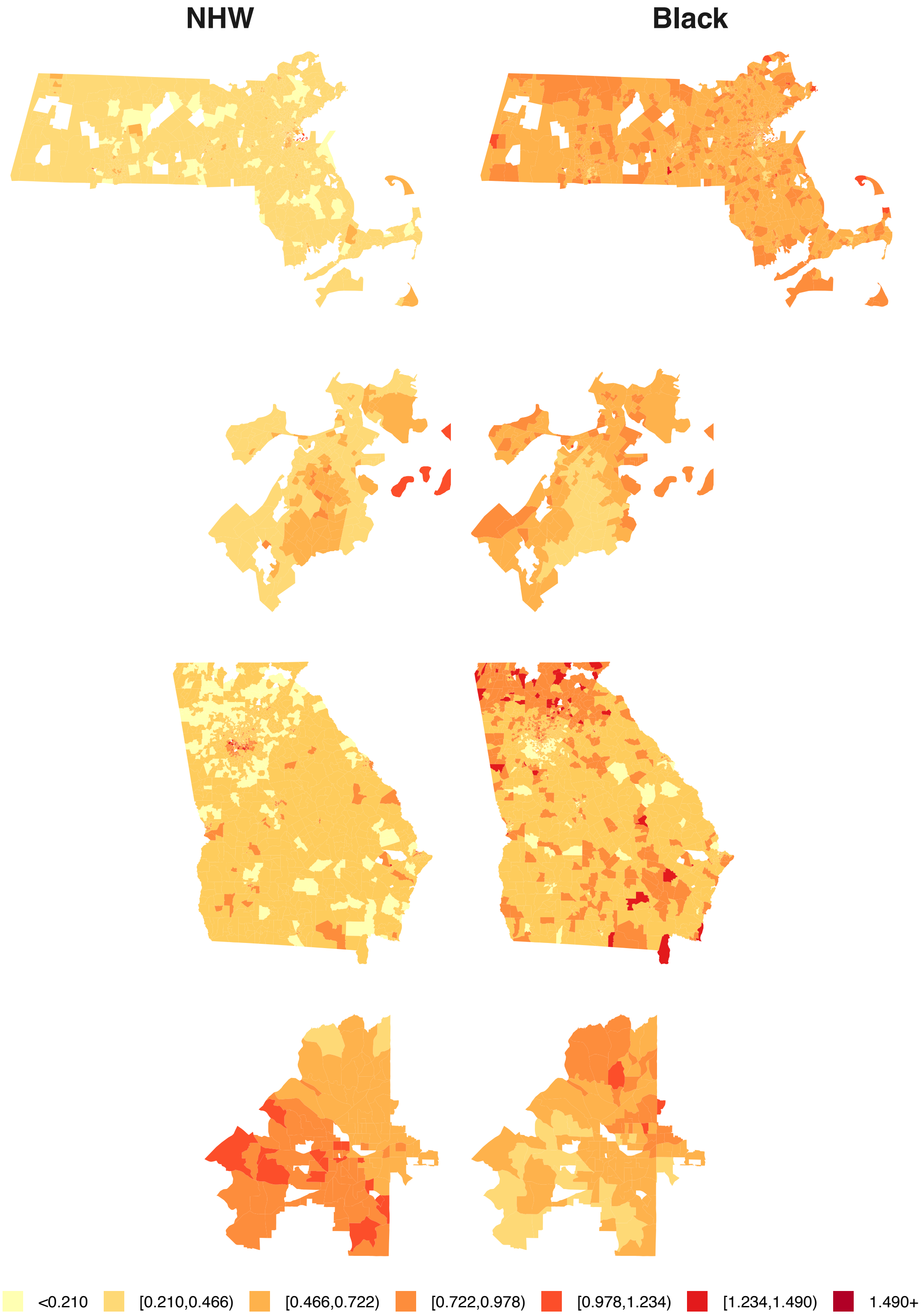

Supplement: Supplementary file 1 — Text S1 Figs. S1 to S5 Tables S1 to S8 [file sciadv.ade8888_sm.pdf]
